# Investigating the utility of ‘global’ measures to quantify neuroimaging biomarkers across the Alzheimer's Disease trajectory

**DOI:** 10.1002/alz70856_103293

**Published:** 2025-12-24

**Authors:** Rifa Sanjida Punnota, Paul Edison

**Affiliations:** ^1^ Imperial College London, London, Greater London, United Kingdom; ^2^ Cardiff University, Wales, United Kingdom

## Abstract

**Background:**

Alzheimer's Disease (AD) is characterized by β‐Amyloid (Aβ) and Tau deposition in brain regions essential for memory, learning, and cognition, followed by glucose hypometabolism and widespread brain atrophy^1^. As such, these pathological processes are widely studied to assess their progression across disease stages. Notably, most studies rely on predefined region‐of‐interest (ROI) analysis, which has several limitations^2^, including potential false negatives from normalizing high and low uptake regions. We investigated the utility of novel ‘global’ measures, designed to mitigate the limitations of ROI analysis, to quantify Aβ, Tau, Glucose Hypometabolism and Grey Matter (GM) atrophy across the AD trajectory.

**Method:**

We analyzed 2596 scans from 743 participants, including [18F]AV1451‐PET, [18F]AV45‐PET/[18F]FBB‐PET, [18F]FDG‐PET, and 3‐Tesla MRI. Standardized Uptake Value Ratio (SUVR) and Voxel‐Based Morphometry (VBM) images were generated, followed by ROI sampling. The single‐subject voxel‐wise analysis compared each scan to 50 controls, yielding 'global' measures from t‐statistical maps, namely: Global Cortical Tau Load, Global Cortical Aβ Load, Global Hypometabolism, and Global GM Atrophy. Using multivariate regression, we then evaluated whether global measures perform better than traditional ROI measures in predicting cognitive function, as measured by ADASCog‐13 scores.

**Result:**

The results revealed that 'global' measures significantly predicted baseline cognition. Global Cortical Tau Load was a better predictor of cognition than medial temporal lobe ROI and performed comparably to whole brain ROI. Global Hypometabolism and Global GM Atrophy were stronger predictors than Whole Brain ROI in MCI and AD participants. Among CN Aβ‐positive individuals, Global Cortical Tau Load emerged as the strongest predictor of baseline cognition.

**Conclusion:**

Our results suggest that ‘global’ measures for Tau, Hypometabolism, and GM Atrophy better predict cognition compared to traditional methods, and these measures can potentially be adopted to quantify these biomarkers in future clinical trials. Global Cortical Tau Load may be a superior method to quantify Tau deposition early in the disease trajectory.

**References**

1. Gulisano W et al. Role of Amyloid‐β and Tau in Alzheimer's. J Alzheimers Dis. 2018;64(S1):S611–31.

2. Gentili C et al. The case for preregistering ROI analyses in neuroimaging. Eur J Neurosci. 2020.

